# The Genetic Diagnostics of Hemochromatosis: Disparities in Low- Versus High-Income Countries

**DOI:** 10.7759/cureus.64074

**Published:** 2024-07-08

**Authors:** Sol Villa Nogueyra, María F Trujillo Rodríguez, María L Garcia Oliva, Andrea Vidal-Gallardo, Amanda Ramírez Leal, Jose Beltran Hernandez, Andres Manuel Vargas Beltran, José D Guillen Sandoval, David Arriaga Escamilla, Marily Martinez Ramirez

**Affiliations:** 1 General Practice, Universidad de Buenos Aires, Buenos Aires, ARG; 2 General Practice, Universidad Nacional Autonoma de Mexico, Mexico City, MEX; 3 General Practice, Universidad de los Andes, Merida, VEN; 4 Faculty of Medicine, Universidad Nacional Autónoma de México, Mexico City, MEX; 5 General Practice, Centro de Estudios Universitarios Xochicalco, Mexicali, MEX; 6 General Practice, Benemerita Universidad Autonoma de Puebla, Puebla, MEX; 7 Internal Medicine, Universidad Justo Sierra, Mexico City, MEX; 8 Internal Medicine, Universidad Nacional Autonoma de Mexico, Mexico City, MEX

**Keywords:** high-income countries, low-income countries, access, genetic diagnostic approaches, hereditary hemochromatosis (hh)

## Abstract

This study provides a comprehensive overview of hereditary hemochromatosis (HH), a genetic condition characterized by iron overload due to excessive iron absorption. It elucidates diverse inheritance patterns and clinical manifestations by exploring mutations in critical genes such as HFE (hemochromatosis), HJV (hemojuvelin), HAMP (hepcidin antimicrobial peptide), TfR2 (transferrin receptor 2), and FP (ferroportin). The significance of early screening, diagnosis, and personalized management strategies based on genetic classification is emphasized, particularly in terms of high-income vs. low-income countries. Addressing challenges in diagnosis, genetic testing accessibility, and healthcare disparities, the study highlights the importance of early detection, cost-effective screening strategies, and enhancing healthcare outcomes globally.

Advanced genetic testing in high-income countries facilitates early diagnosis and management, reducing complications such as liver disease and cardiomyopathy. In contrast, low-income populations face several barriers, including limited access to genetic testing, high costs, and inadequate healthcare infrastructure. Cost-effective serum ferritin (SF) and transferrin saturation (TS) tests and emerging point-of-care (POC) tests offer affordable diagnostic options for low-resource settings. Additionally, the ongoing development of hepcidin measurement methods holds promise for enhancing diagnostic capabilities. Implementing these strategies can aid healthcare providers in improving global HH management and reducing the burden of iron overload complications. Furthermore, the study underscores the need for public health initiatives to raise awareness about HH, promote routine screenings, and advocate for equitable healthcare policies.

Collaborative efforts between governments, healthcare organizations, and research institutions are crucial in addressing the global burden of HH. By fostering international cooperation and resource-sharing, it is possible to bridge the gap between high-income and low-income countries, ensuring all individuals have access to the necessary diagnostic and treatment options. This holistic approach can ultimately lead to better health outcomes and improved quality of life for individuals affected by HH worldwide. This comprehensive examination of HH not only illuminates the genetic and clinical aspects of the condition but also provides a roadmap for addressing the multifaceted challenges associated with its diagnosis and management.

## Introduction and background

Hereditary hemochromatosis (HH) is a genetic disorder characterized by excessive iron absorption due to low levels of hepcidin, a hormone regulating iron. Mutations in five key genes - HFE (hemochromatosis), HJV (hemojuvelin), HAMP (hepcidin antimicrobial peptide), TfR2 (transferrin receptor 2), and FP (ferroportin) - are responsible for HH. These mutations are predominantly inherited in an autosomal recessive pattern, except for FP, meaning a person needs to inherit two defective copies to be affected. Mutations in FP are inherited in an autosomal dominant pattern, where a single defective copy is sufficient to cause the condition [1,2]. Given the different types of mutations mentioned, a classification for HH has been established to differentiate between its inheritance pattern, iron accumulation, and organ damage, predominant cell distribution of iron within the liver, hepcidin production, response to phlebotomy treatment, and the onset of symptoms within the age of presentation [2,3]. This classification of HH is essential for medical practice and research as it helps differentiate between genetic mutations associated with the condition. This classification aids in predicting the severity of iron accumulation, organ damage, treatment response, and symptom onset age.

By identifying specific mutations, healthcare providers can tailor treatment plans to enhance early detection and personalized management strategies for at-risk individuals, ultimately leading to improved awareness and prevention of severe complications. HH is a genetic condition where the body absorbs too much iron, leading to iron overload. Clinical manifestations of HH depend on the timing of diagnosis. Early stages can be asymptomatic, leading to increased prevalence with age, particularly in males, who are affected two to three times more often than females [1]. HFE mutations are most common in individuals of northern European descent. They can be associated with other conditions causing liver damage, such as hepatitis C infection and alcoholic or non-alcoholic liver disease. HJV and HAMP mutations can occur in any population, while TfR2 mutations are more frequent in individuals of southern European and Japanese descent [1,2]. Although rare, HJV and HAMP mutations often present in individuals under 30. In such cases, liver complications and extrahepatic issues like cardiomyopathy, hypogonadotropic hypogonadism, and amenorrhea may indicate HH in younger adults [1,3]. 

Genetic diagnosis is recommended for patients with elevated transferrin saturation (TS) and high serum ferritin (SF) levels that other conditions cannot explain. However, diagnosing HH in low-income populations is challenging due to limited access to genetic testing, high costs, and inadequate healthcare infrastructure, which will be explored in this review. This review demonstrates how early screening is indicated for the populations at risk or patients with suggestive clinical presentation and the differences between the approaches of low and high-income countries toward screening methods. Although healthcare systems in underdeveloped countries may lack several screening methods due to high costs or delayed manifestations and suspicion of the different subtypes of HH, measures should be taken to raise awareness among primary care providers, specialists, and even the patient population [4].

## Review

Genetic basis and diagnosis of hemochromatosis

Five genes have been identified as causative factors in HH, including HFE, HJV, HAMP, and TfR2, which follow an autosomal recessive pattern, and the FP gene, which can be inherited in an autosomal dominant pattern [5].

Type 1 HH: HFE Gene Mutations

Homozygous C282Y mutations in the HFE gene primarily cause type 1 HH. Less commonly, individuals may have compound heterozygosity for C282Y and H63D mutations [5,6]. HFE is a protein membrane comprising proteins that capture the signal medium and trigger chemical and physical mechanisms within the cell, affecting the communication between themselves and other cells. The role of HFE is to regulate the interaction of the transferrin receptor with transferrin. In the case of mutated HFE protein, the mutant protein cannot reach the cell surface and remains trapped intracellularly, leading to this regulatory feature being missing [5-7]. The impairment of transferrin's entry into crypt cells is thought to lead to a state of intracellular iron deficiency, which affects the cellular signals and leads to an increased expression of proteins responsible for iron absorption in the cellular membrane, leading to high iron levels from these cells after differentiation into villus enterocytes [5,6].

Type 2 HH: HJV and HAMP Gene Mutations

Mutations in genes responsible for encoding HJV or Hepcidin lead to type 2 HH, specifically HJV mutations resulting in type 2A and HAMP mutations resulting in type 2B. These genetic alterations lack functional hepcidin and are linked to significant iron accumulation that occurs earlier than type 1. G71D and R59G have been exclusively observed in the heterozygous form of this condition [8,6].

Type 3 HH: TfR2 Gene Mutations

Six TfR2 gene mutations have been identified for type 3 HH, which are exceptionally rare and predominantly observed in Southern European populations. These mutations result in a faster rate of iron buildup compared to other kinds of HH. The mechanism by which mutations resulting in the loss of function of this receptor contribute to iron accumulation remains unclear. Ongoing research is crucial to elucidate these mechanisms and improve therapeutic approaches for HH. This can lead to better management of iron overload and its associated complications, ultimately enhancing patient outcomes and quality of life [9].

Type 4 HH: FP Gene Mutations

Type 4 HH arises from mutations in the FP gene, with at least a dozen pathogenic mutations. There is a distinct alteration in the pattern of iron distribution within the liver, characterized by iron in reticuloendothelial cells, resulting in poor tolerance to phlebotomy. Mutations in FP could represent a significant cause of iron overload among individuals of African American descent [10,11]. The characteristics of all four types of HH are summarized in Table 1, including gene classification, inheritance, chromosomal location, and function.

**Table 1:** Gene classification of hereditary hemochromatosis The gene responsible for each subclassification of hemochromatosis [5-11] Hemochromatosis (HFE). Long arm (q). Short arm (p)

Gene name	Classification	Inheritance	Function	Chromosomal location
HFE	Type 1	Autosomal recessive	Transferrin receptor interaction, uptaking of transferrin-bound iron	6p21.3
Hemojuvelin	Type 2	Autosomal recessive	Hepcidin expression regulation	1q21
Hepcidin	Type 2	Autosomal recessive	Reduced release of iron by macrophages and enterocytes	19q13.1
Transferrin receptor 2	Type 3	Autosomal recessive	Absorption of iron by hepatocytes	7q22
Ferroportin	Type 4	Autosomal dominant	Exporting iron from macrophages and enterocytes	2q32

Clinical manifestations of hemochromatosis

The condition commonly presents in middle-aged female patients; the clinical manifestations include arrhythmias, heart failure, hyperpigmentation, hypothyroidism, diabetes, hepatomegaly, and some biochemical alterations such as mild abnormalities in the liver profile, which include aspartate aminotransferase (AST), alanine aminotransferase (ALT), alkaline phosphatase (ALP), bilirubin, and albumin determinations [12]. Nevertheless, it is difficult to identify patients with manifestations before the development of advanced organ damage [13,14]. For example, HH and other diseases, such as dengue, could affect the biochemical liver profile, which often causes elevation of the aminotransferases. Nevertheless, the clinical manifestations of dengue are different [12]. Therefore, the diagnostic evaluation of HH relies on the patient's proper clinical and laboratory evaluation [15].

Patients with an unexplained abnormality in the liver profile should be screened for hemochromatosis. Initial screening exams for most patients include serum-transferrin-iron saturation and ferritin [5]. Levels of SF concentration and TS are elevated in individuals with HH. Additional assessments are advisable if ferritin levels exceed 200 µg/L or TSAT exceeds 45%. These tests together have a negative predictive value of 97% for HFE mutations. Serum TS and ferritin concentrations are increased in these patients. Iron overload in the liver can be detected in imaging tests such as MRI, or iron deposits can be seen in hepatocytes on a liver biopsy [16-18].

**Figure 1:**
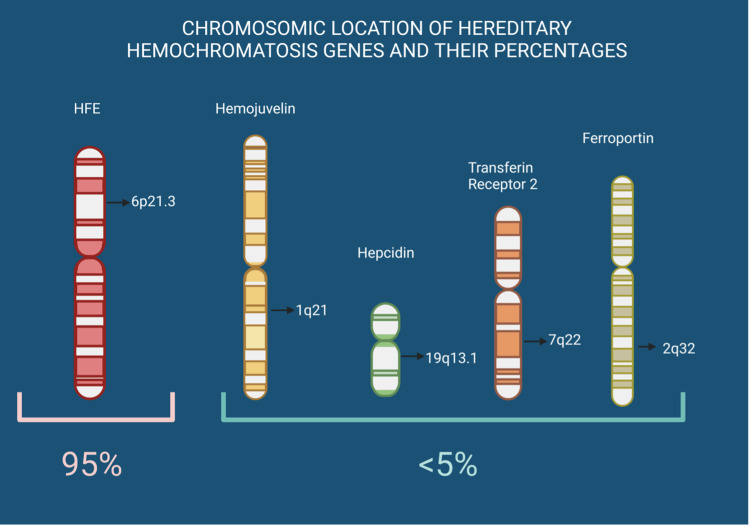
Chromosomic location of hereditary hemochromatosis genes and their proportions Mutations in the HFE gene cause 95% of all hereditary hemochromatosis cases. Hemojuvelin, hepcidin, transferrin receptor 2, and ferroportin gene account for the other 5% [14,15,18]. Credits to María Fernanda Trujillo Rodríguez. Figure created with BioRender Hemochromatosis (HFE). Long arm (q). Short arm (p)

The gold standard for diagnosing HH is a genetic test. Of note, 95% of all cases are caused by mutations in homozygous C282Y and heterozygous C282Y/H63D, so HFE genotyping is recommended. Since the other types of mutations are far less common, genetic testing for non-HFE HH must be considered in low-income communities if there is a family history of iron overload or hepatic iron overload, which imaging can demonstrate. While the penetrance reported varies in different studies and populations, it typically shows a higher occurrence in males and rises with age. These findings pose notable challenges for using genomic screening in high-income communities, a reliable method for detecting HH [13,14,17]. Figure 1 summarizes this information.

Treatment

A patient is a biopsychosocial being who needs to be treated integrally; that is why the approach toward a hemochromatosis patient needs to be focused not only on the iron overload but also on the comorbidities and the diet management [19]. Phlebotomy remains the mainstay of therapy and is used to remove excess iron and maintain low normal body iron stores. It should be initiated in men with SF levels of 300 µg/L or more and women with SF levels of 200 µg/L or more, regardless of the presence or absence of symptoms. Once the decision is made to perform phlebotomy, the initial phase is typically done with weekly removal of 500 mL of blood. Larger volumes of blood, usually 1,000 mL, can be removed if tolerated to expedite the removal of excess iron [13]; conversely, in patients who do not tolerate weekly phlebotomies, smaller volumes of blood can be removed, or the interval between sessions can be increased, although this will lengthen the time required to mobilize the excess iron. It is important to check the hemoglobin level before and during treatment to ensure it is above 11 g/dL. SF level should be checked monthly during phlebotomy until a goal SF level of 50-100 ng/mL is reached [19,20].

After the SF has reached its goal level, the initial induction phase of treatment is complete, and the maintenance phase follows. The goal of this phase is to maintain SF levels near 50 ng/mL, and the frequency of phlebotomy is typically 3-4 times per year [20]. For those patients who are intolerant or refractory to phlebotomy or when phlebotomy has the potential for harm, such as in patients with severe anemia or congestive heart failure, the use of iron chelation for treatment of HH is recommended [20,21]. Iron chelation effectively treats HH in small clinical trials [20,22,23]. Chelation is not recommended as the first-line therapy for HH, given the effectiveness of phlebotomy, the associated side effects of chelation, including hepatic and renal toxicity, and the relatively small sample size of clinical trials supporting chelation [20].

Some new therapies are being developed, such as deferasirox (an iron chelator), which could be a safe alternative to phlebotomy in selected patients [22,20,23]. Most recently, the role of iron and the gut microbiome has been described, suggesting a relationship between these two, opening the discussion and further research in this area [24-26]. As it was mentioned above, patients with HH can present with different clinical manifestations such as liver, pancreas, joint, and heart problems. These comorbidities often require additional, specific management [19]. Dietary management of hemochromatosis includes avoidance of medicinal iron, mineral supplements, excess vitamin C, and uncooked seafood. This can reduce the rate of iron reaccumulation, reduce retention of nonferrous metals, and help reduce complications of liver disease, diabetes mellitus, and Vibrio infection [19].

This comprehensive approach to managing hemochromatosis can decrease the frequency and severity of iron overload, improve quality of life, and increase longevity [19]. There is a need to point out to the patients that the use of herbal products has not been demonstrated to be helpful in this disease; this point takes relevance as we consider that almost 80% of the population in middle-income countries takes herbal medicine [27,28]. Referral for liver transplantation (LT) should be considered in patients with HH with end-stage liver disease or hepatocellular carcinoma (HCC). LT is curative not only in patients with decompensated cirrhosis and HCC, but it also normalizes hepcidin levels and alterations in iron metabolism [15,20].

Comparative analysis of disparities in diagnostic capabilities

Understanding disparities in diagnostic capabilities is crucial for improving global health outcomes. Effective diagnostic tools are essential for accurate disease identification and treatment, impacting patient care and public health. In regions with limited diagnostic capabilities, the lack of accurate and timely diagnoses can lead to higher morbidity and mortality rates, hinder disease control efforts, and exacerbate health inequities. Addressing these disparities ensures that all populations, regardless of geographic location or economic status, have access to necessary healthcare services, promoting health equity and enhancing global health security [29-32]. The World Bank classifies economies based on Gross National Income (GNI) per capita. Low-income economies, with a GNI per capita of $1,025 or less, and high-income economies, with a GNI per capita of $12,376 or more, provide a framework for understanding economic disparities that impact diagnostic capabilities and health outcomes across regions [33,34].

Resource constraints significantly impact the early identification and treatment of different diseases and HH, often leading to underdiagnosis and severe complications if untreated, as has been pointed out several times before [35-37]. Socioeconomic factors influence HH diagnosis and management, underscoring the need for population screening programs to reduce undiagnosed cases [38]. A two-tiered screening strategy can be effective in high-risk populations-initial laboratory tests followed by confirmatory diagnostics [38]. Liver biopsy demonstrated a sensitivity of 96% and specificity of 100% for detecting HH [39-41]. DNA testing for HH gene mutations, particularly the C282Y mutation, demonstrates variable levels of sensitivity and specificity.

TS has been shown to have a sensitivity of 75% and a specificity of 82% [42,43], making it a screening rather than a diagnostic test. Screening with confirmation of the diagnosis by liver biopsy costs between $5,079 and $8,813 per case detected (excluding administrative costs), depending on the screening strategy. If a DNA test were used instead of a liver biopsy, the cost would be reduced to $3,954-$4,410 per case. This reduces to $2,457 by detecting additional cases by screening family members.

**Figure 2:**
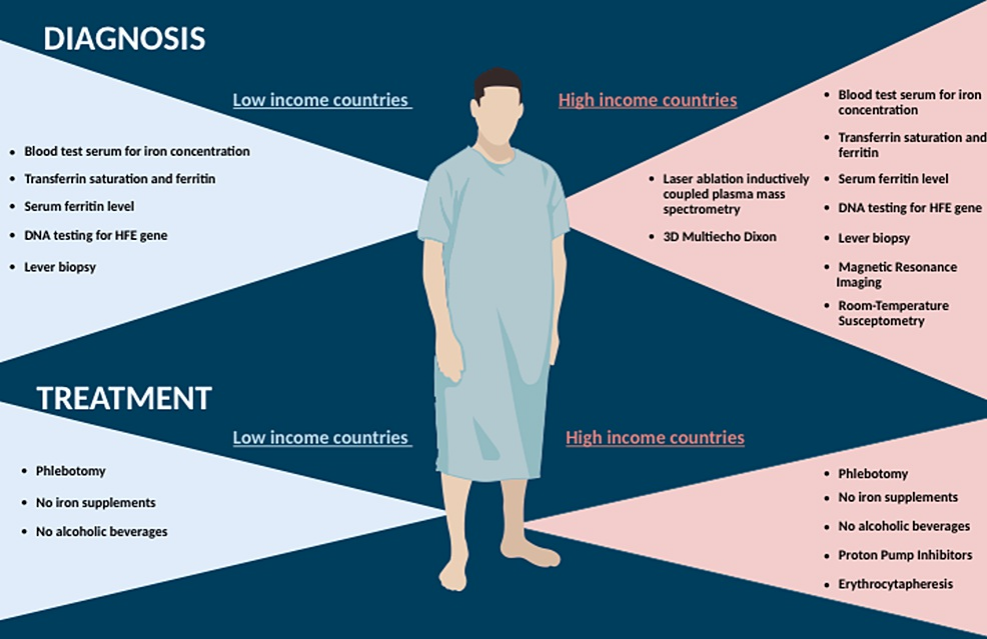
Diagnosis and treatment The diagnosis and treatment of hereditary hemochromatosis generally match in both cases, but within high-income countries, we can find a more varied and advanced range to address this disease [38-41,44,45,49]. Deoxyribonucleic acid (DNA). Hemochromatosis (HFE)

TS screening is cost-effective, costing around $11 in India and $608 in the US, and can reduce complications through early detection and treatment [44,45]. The most cost-effective strategies utilized TS for initial screening, followed by DNA testing. Reduction in the cost of TS would lead to a significant decrease in total screening costs [46]. The research underscores the significance of early detection by illustrating the cost-effectiveness of screening compared to no screening [40,47]. For instance, Denmark's early diagnosis through population screening by measuring ferritin and TS through the biochemical routine analysis package has proven cost-effective in managing HH among 500,000 affected individuals out of a five million population [48,49]. More information about the diagnosis and treatment differences is presented in Figure 2.

As previously stated, low-income countries have a GNI per capita of $1,025 or less [50]. For example, in Afghanistan, insufficient resources and allocation techniques lead to issues like unnecessary transfusions that can cause adverse effects and organ damage like cirrhosis, HCC, cardiomyopathy, arthropathies, etc. [51,52]. Early diagnosis utilizing laboratory screening tests may reduce morbidity and healthcare expenses [53]. The annual healthcare costs in patients with type 2 diabetes, hypertension, arthritis, and chronic kidney disease with HH were $6,968, $7,424, $2,967, and $43,847, respectively, higher than type 2 diabetes, hypertension, arthritis, and CKD patients without HH [39]. Additionally, the complexity of genetic testing beyond HFE may pose challenges due to financial, structural, and scheduling constraints, necessitating hepatic iron measurement before such procedures [54].

However, screening in healthcare systems may encounter obstacles due to variations in costs, procedures, and resource prioritization, necessitating comprehensive cost-effectiveness models [55]. Addressing these aspects through simplified services, consistent monitoring, and patient-focused clinics can enhance HH care in diverse cultural and socioeconomic contexts. Optimizing resource allocation in primary health centers is crucial for decreasing healthcare inequities and enhancing diagnostic efficiency. Improving screening procedures, raising awareness, and ultimately enhancing clinical outcomes for HH patients hinge upon addressing these discrepancies. Establishing such initiatives globally requires robust health economic evidence. However, screening in healthcare systems may face obstacles due to variations in costs, procedures, and resource prioritization, necessitating comprehensive cost-effectiveness models. Addressing these issues through simplified services, consistent monitoring, and patient-focused clinics can enhance HH care in diverse cultural and socioeconomic contexts [56].

Primary care providers must be educated and updated about HH diagnosis and treatment to provide comprehensive health information to patients. This requires collaboration among organizations, health educators, and physicians, even when differences in medical provider education need to be addressed to create educational programs and advertising campaigns to support the HH patient community [50,57].

Hemochromatosis genetic diagnostics in high-income countries: availability and access

High-income countries have a GNI per capita of $12,376 or more [58]. Diagnosis of HH often starts with identifying elevated SF levels. Specific diagnostic tests, including stepwise biochemical and genetic analyses, are available to ensure accuracy. However, these tests should not be administered universally to all patients with elevated SF levels but should be reserved for those with a high suspicion of HH or a documented family history [59,3]. The diagnosis of HH is challenging; the presence of elevated SF levels can also indicate other conditions such as chronic liver disease, sepsis, acute liver disease, dengue, and systemic inflammatory conditions. Therefore, it is crucial to systematically exclude alternative etiologies before considering HH [59,60].

Elevated SF levels vary by gender: greater than 300 ng/dL in males and greater than 200 ng/dL in females. If SF levels exceed 45%, further testing for HH should include genetic analyses for common HFE gene mutations like C282Y and H63D. Initially, biochemical tests such as serum TS are recommended due to the high costs of genetic tests [59,60]. As mentioned in previous sections, the cost of screening with confirmation of the diagnosis by liver biopsy costs between $5,079 and $8,813 per case detected (excluding administrative costs). If a DNA test were used instead of a liver biopsy, the cost would be reduced to $3,954-$4,410 per case. This reduces to $2,457 by detecting additional cases by screening family members [46].

As we observed, a high-income country can approach the costs regardless of the chosen diagnostic option. Nevertheless, the most cost-effective strategy is to utilize TS for initial screening, followed by DNA testing [46].

Follow-up of the Disease

Different tools could be used for the diagnosis and follow-up of HH, such as imaging. Ultrasound (US) scan of the liver is often the first diagnostic step when the patient presents with elevated liver enzymes or is suspected of liver disease. Ultrasound cannot detect iron in liver tissue and, therefore, cannot be used for the diagnosis of iron overload in hemochromatosis, but is useful for differential diagnostic purposes to exclude other causes of elevated liver enzymes and non-alcoholic fatty liver disease (NAFLD) [61,62]. Ultrasound is a safe, low-cost, non-invasive, portable imaging modality that is easy to reproduce and yields excellent results when performed by a trained operator [63].

Ultrasound is the method for evaluating different clinical situations in experienced hands and is performed with dedication, exhaustively, and responsibly. Sometimes, it is the first, the most appropriate, and sometimes the only imaging technique capable of solving the diagnostic challenge. Its only disadvantage is being a dependent operator since it requires continuous training and supervision from the beginning by someone experienced [63]. As the benefits of ultrasound technology become apparent, efforts to integrate ultrasound into pre-clinical medical education are growing. Still, we need to consider the economic country burden that causes the creation of medical staff with the skills to use ultrasound appropriately [64,65]. Furthermore, several techniques are employed to diagnose iron overload, such as magnetic resonance imaging (MRI) and computed tomography (CT), which are the most promising non-invasive ones [66]. Computed tomography (CT) of the liver can detect iron in the liver parenchyma, but the method requires special programming of the scanner, is semi-quantitative, and has several sources of error [67].

MRI scan of the liver is, in the experienced setting, a good method for quantitative measurement of the liver iron content. Hepatic iron causes loss of signal intensity in the liver, which increases proportionally to the amount of iron deposition. The hepatic iron is then quantified by measuring the ratio of the signal intensity of the liver with that of a reference tissue (e.g., paraspinal muscle) [20,68-71]. MRI is a rapid, non-invasive, and cost-effective technique that could limit the use of liver biopsy to assess liver iron content [61]. High-income countries have the power to access and afford an HH patient's costs, as previously mentioned. Nevertheless, the main focus should be strategies that allow earlier diagnosis of HH that can improve patient outcomes and reduce healthcare costs [72].

Hemochromatosis genetic diagnostics in low-income countries: challenges regarding access and resources

Low-income countries face healthcare challenges, including lacking essential infrastructure, laboratory supplies, basic equipment, skilled personnel, supply chain management, and maintenance. Additionally, there is a reliance on empirical treatment, inadequate quality management systems, and a lack of government standards for laboratory testing. These issues result in delayed or inaccurate diagnoses and ineffective treatments [73,74]. HH can be highly suspected using biochemical tests but needs confirmation with genetic tests. Serum TS, SF, and unsaturated iron-binding capacity (UIBC) are biochemical tests used for diagnosis, which can be further confirmed with HFE genotyping [4].

Genetic screening for HH involves detecting specific mutations using techniques like polymerase chain reaction (PCR) and restriction fragment length polymorphism (RFLP) [75,76]. As mentioned in previous sections, the most cost-effective strategy is to utilize TS for initial screening, followed by a genetic test [46]. It has been demonstrated that early detection reduces morbidity and, ultimately, healthcare costs for low-income individuals and countries [53]. Patients with HH imply a higher healthcare cost compared with non-hemochromatosis patients [50,53]. Insufficient resources and allocation techniques lead to unnecessary use of medical resources and complications in HH patients. Also, these include more inpatient admissions, emergency department visits, outpatient visits, and pharmaceutical prescriptions, which in the long run is less cost-effective for a country [50,51,56].

Early detection is a significant challenge in low-income countries, but various strategies can be developed to address this [73,74]. Creating national laboratory policies and strategic plans, as implemented by Ethiopia in 2005 for HIV serology, chemistry, and hematology, can be effective [73]. Establishing public-private partnerships (PPPs), such as the Pfizer Global Health Fellows program from 2003 to 2005, can strengthen health systems in low-income countries by providing technical assistance and improving organizational performance [77]. Strengthening the interface between diagnostics and clinicians can lead to better uptake and use of laboratory test results [74]. Implementing a laboratory medicine leadership program, similar to the US Centers for Disease Control and Prevention’s Field Epidemiology Training Program, can enhance collaboration between clinicians and laboratorians [78]. Furthermore, a study on "Haemochromatosis patients' research priorities" indicates that better-informed doctors are crucial for improved HH disease management. Focusing on strategies that allow earlier diagnosis of HH can improve patient outcomes and reduce healthcare costs [72].

Recommendations

To address these disparities, it is essential to implement cost-effective diagnostic alternatives such as SF and TS tests, emerging point-of-care (POC) tests, and hepcidin measurement methods. Additionally, international collaboration and support are crucial to enhance healthcare infrastructure and training in low-income countries. Enhancing healthcare infrastructure, improving clinical assessment training, and increasing the availability of affordable diagnostic technologies are critical steps toward improving HH's early detection and management in low-resource settings. Future research should focus on developing scalable and affordable diagnostic solutions tailored to these environments. By adopting a multifaceted approach, healthcare providers can significantly enhance global HH management and reduce the burden of iron overload complications.

## Conclusions

The disparities in genetic diagnostics for HH between low and high-income countries represent a significant global health challenge. While advanced genetic testing in high-income countries enables early diagnosis and management, thereby reducing severe complications, low-income populations face substantial barriers, including limited access to testing, high costs, and inadequate healthcare infrastructure.
